# Comparison of Laparoscopic Appendectomy with open appendectomy in Treating Children with Appendicitis

**DOI:** 10.12669/pjms.322.9082

**Published:** 2016

**Authors:** Guoqing Yu, Aihua Han, Wenjuan Wang

**Affiliations:** 1 Guoqing Yu, Pediatric Surgery Department, Binzhou People’s Hospital, Shandong, China; 2 Aihua Han, Pediatric Surgery Department, Binzhou People’s Hospital, Shandong, China; 3 Wenjuan Wang, Psychological Rehabilitation Department, Binzhou People’s Hospital, Shandong, China

**Keywords:** Appendicitis, Laparoscopic appendectomy, Open appendectomy, Clinical effect

## Abstract

**Objective:**

To analyze feasibility and curative effect of laparoscopic appendectomy in the treatment of pediatric appendicitis and compare it with open appendectomy.

**Methods:**

Two hundred and sixty patients were selected for this study and randomly divided into open appendectomy group (130 cases) and laparoscopic appendectomy group (130 cases). Patients in open appendectomy group underwent traditional open appendectomy, while patients in laparoscopic appendectomy were treated with laparoscopic appendectomy. Incision length, blood loss during operation, duration of operation, time to leave bed, anus exhausting time, time to take food, catheter drainage time, urinary catheterization time, time of using antibiotics, use of pain killer and incidence of complications such as incision infection, residual abscess and intestinal obstruction were compared between two groups.

**Results:**

We found relevant indexes including length of incision, amount of bleeding and duration of operation in laparoscopic appendectomy group were better than open appendectomy group after surgery; and differences were statistically significant (P<0.05). Indexes such as time to out of bed, time to take food, exhaust time, drainage time, catheterization time and application time and use of antibiotics in laparoscopic appendectomy group were all superior to open appendectomy group, and differences had statistical significance (P<0.05). Incidence of complications in laparoscopic appendectomy group was much lower than open appendectomy group and the difference was statistically significant (P<0.05).

**Conclusion:**

Laparoscopic appendectomy has advantages of small trauma, sound curative effect, low incidence of complications and rapid recovery and can effectively relieve pain of children suffering from appendicitis. Hence it is worth promotion and should be preferred.

## INTRODUCTION

Previous clinical treatment mostly used open appendectomy which has been practiced for more than 100 years as a classical therapy for treating pediatric appendicitis. But now this therapy is gradually rejected by patients and their family members due to problems such as incision infection, wound infection, intestinal adhesion, intestinal obstruction and slow recovery of intestinal tract.^1^,^2^ With the development of medical technologies and improvement of medical equipment’s, clinical researches on treating infantile appendicitis with laparoscopic surgery has become more wide spread.^3^ In laparoscopic surgery, abdominal organs are photographed by image pickup system under the illumination of cold light source and displayed on screen; doctors operate under the guidance of screen. Laparoscopic surgery characterized by small trauma and damage can reduce or even avoid traction and turnover of intestinal tube.

But laparoscopic surgery in children fails to be widely accepted due to various reasons. Experts and scholars in China and oversea disputes selection of surgical method.^7^ Some scholars hold the opposite views. For instance, Ma GQ believed that, laparoscopic surgery has not been able to completely replace open appendectomy and has mentioned some conditions in which it was not suitable for laparoscopic surgery including patients who had stomachache for more than 72 hours, had inflammatory mass in local appendix or abscess around appendix, had serious adhered appendix and surrounding organs, patients with obstructive factors such as history of complex surgery in abdomen, important organ dysfunction or non-function and abnormal coagulation function and patients with pregnancy complicated with appendicitis.^4^ Hu WL, et al. thought that with laparoscopic appendectomy, overall, cost more compared to open appendectomy.^5^ But most scholars believe that laparoscopic appendectomy which is safe and effective had outstanding advantages like small incision, infection rate, blood loss during operation, postoperative recovery time, postoperative complication and cosmetic result.^6^,^7^

This study retrospectively analyzed 260 patients of pediatric appendicitis who received treatment in Binzhou People’s Hospital, Shandong, China between January 2013 and November 2014 and compared the curative effect of laparoscopic appendectomy and open appendectomy in treating acute appendicitis. Through observing differences of clinical indexes such as blood loss during operation, duration of operation, time to leave bed, anus exhausting time, time to take food, catheter drainage time, urinary catheterization time, time to recovery of gastrointestinal function, length of stay, etc., we have discussed the advantages and disadvantages of laparoscopic appendectomy and open appendectomy.

## METHODS

We selected 260 children who suffered from appendicitis and received treatment in department of pediatric surgery in Binzhou People’s Hospital between January 2013 and November 2014. All patients had been confirmed having appendicitis according to diagnostic standard of appendicitis released by WHO^8^ and failed to be cured by conservative treatment. They were found with symptoms of fever, nausea, emesis, abdominal distension, stomachache, rebound tenderness on right lower quadrant and tenderness and proved to have seroperitoneum by ultrasonography. All patients were randomly and evenly divided into laparoscopic appendectomy group and open appendectomy group. In open appendectomy group, there were 72 males and 58 females with age ranging from 6 to 12 years (average 7.20±1.50 years); 14 had chronic appendicitis, 98 developed acute appendicitis and 18 had acute purulent appendicitis. In laparoscopic appendectomy group, there were 82 males and 48 females with age ranging from 5 to 12 years (average 7.90±1.40 years); 18 cases had chronic appendicitis, 100 cases suffered from acute appendicitis and 12 cases had acute purulent appendicitis. No statistically significant difference was found in age, gender and distribution of appendicitis category; therefore, results were comparable.

### Traditional open appendectomy

Patients were given either general anesthesia or epidural anesthesia. A trans rectal incision was cut at mcburney point. Sroperitoneum was removed. Msentery and vermiform appendix were processed as usual. Apendiceal stump was embedded with purse string suture or figure-of-eight suture. Abdominal cavity was washed by metronidazole. Abdominal exudatives was wiped away with wet saline gauze. After being sutured layer and layer, the incision was washed by Povidone-iodine solution. Drainage tube was inserted if appendix perforates, and removed according to volume of drainage and body temperature.

### Laparoscopic appendectomy

Laparoscopic appendectomy was performed under tracheal intubation and general anesthesia. A 5 mm incision was made on inferior margin of umbilicus. CO^2^ pneumoperitoneum was made and then 5 mm canula sheath and laparoscope were inserted. Two incisions were made at the position of equilateral triangle formed by left inguinal region and umbilicus under laparoscope; and 3 mm or 5 mm canula sheath was inserted.^10^ Peritoneal fluid was absorbed when patients raised legs 15 degrees higher than head and inclined to left side for 15 to 30 degrees. Appendix was separated from adhesion. Mesoappendix was divided with ultrasound knife till appendix root. For those who had appendiceal stump perforation or body perforation, appendix root was double ligatured with absorbable clips or No.7 suture line, and appendiceal stump mucous membrane was processed by electrocoagulation. For those who could not undergo ligation due to appendix root perforation or gangrene, appendiceal stump was processed by figure-of-eight suture and reinforced by omentum majus. Appendix was packed into specimen bag or taken out along with cannula sheath. Abdominal cavity was washed constantly until the liquid become clear. The incision was disinfected with povidone-iodine solution and the skin was closed using band-aid. Drainage tube was inserted into pelvic cavity if abdominal cavity was seriously polluted.

### Observation index

Observation indexes included duration of operation (time from cut on skin to skin suture, minutes), length of incision (total length of incision cut on skin, mm), amount of bleeding (blood loss during operation, ml), time to out of bed, time to take food, exhaust time, catheterization time (day), application time of antibiotics (day from the ending of operation to withdrawal of antibiotics), usage of analgesic (the proportion of patients taking pain killer after surgery), drainage time (time from insertion of abdominal cavity drainage-tube to removal of tube, day) and incidence of complications (probability of having wound infection, intestinal obstruction, intra-abdominal abscess, etc., %).

### Statistical method

SPSS19.0 software package was used to process data. Measurement data were expressed as mean ± SD. Comparison between groups was performed using t test. Enumeration data were expressed as no. of cases (n). Difference of rate (%) between groups was compared by chi-square test. Difference was considered to be statistically significant if P<0.05.

## RESULTS

### Comparison of clinical indexes

Compared to open appendectomy group, laparoscopic appendectomy group performed better in clinical indexes such as length of incision, amount of bleeding and duration of operation. As to the length of incision, the average value of open appendectomy group was 7.37±3.00 cm, 3.5 cm the shortest and 12 cm the longest; the average value of laparoscopic appendectomy group was 2.11±0.21 cm, 2.0 cm the shortest and 2.5 the longest. A significant difference was observed between two groups (P<0.05). As to amount of bleeding, the average value of open appendectomy group was 27.70±38.20 mL, 2 ml the most and 200 mL the least; the average value of laparoscopic appendectomy group was 7.94±7.53 mL, 1 mL the least and 50 mL the most. There was also a remarkable difference (P<0.05). As to duration of operation, the average value of open appendectomy group was 108.06±51.47minutes, 25 minute the shortest and 220 minutes the longest; the average value of laparoscopic appendectomy group was 63.48±27.46 minute, 25 minute the shortest and 150 minues the longest. The difference was statistically significant. The detailed comparison is shown in Table-I.

**Table-I:** Comparison of clinical indexes between two groups.

Group	Length of incision (cm)	Volume of bleeding (ml)	Duration of operation (min)
Open appendectomy group	7.37±3.00	27.7±38.2	108.06±51.47
Laparoscopic appendectomy group	2.11±0.21	7.94±7.53	63.48±27.46
T value	10.19	2.931	4.648
P value	0.000	0.04	0.000

### Comparison of curative effect

Compared to open appendectomy group, laparoscopic appendectomy group had better curative effect indexes including time to out of bed, time to take food, exhaust time, drainage time, catheterization time and time to take antibiotics. The difference was statistically significant (P<0.05). Detailed data are shown in Table-II.

**Table-II:** Comparison of clinical effect indexes between two groups.

Group	Time to out of bed (day)	Time to take food (day)	Time to exhaust (day)	Drainage time (day)	Catheterization time (day)	Time to take antibiotics (day)
Open appendectomy group	2.71±1.32	3.38±1.23	3.05±1.02	2.35±2.70	1.49±1.19	6.51±4.23
Laparoscopic appendectomy group	1.71±0.63	2.12±0.70	2.00±0.66	1.12±2.26	0.61±0.66	3.88±1.76
T value	4.154	5.450	5.333	2.254	3.981	3.420
P value	0.000	0.000	0.000	0.026	0.000	0.001

### Comparison of usage rate of analgesic

Number of cases taking analgesic in open appendectomy group and laparoscopic appendectomy group after operation was 16 and 8 (12.30% vs 6.10%). Usage of analgesic in laparoscopic appendectomy group was lower than open appendectomy group, but no significant difference was observed. Detailed data are shown in Table-III.

**Table-III:** Comparison of usage rate of analgesic between two groups [n(%)]

Group	No. of cases taking analgesic	No. of cases not taking analgesic
Open appendectomy group (n=130)	16 (12.3)	114 (87.7)
Laparoscopic appendectomy group (n=130)	8 (6.1)	122 (93.9)
X^2^ value	0.375	
P value	>0.05	

### Incidence of complications

In open appendectomy group, 16 cases developed complications (12.31%), including 8 cases of wound infection (6.15%), 2 cases of adhesive intestinal obstruction (1.54%) and 6 cases of intra peritoneal abscess (4.62%). In laparoscopic appendectomy group, only 6 cases were found with complications (4.62%), including two cases of wound infection (1.54%) and four cases of intraperitoneal abscess (3.08%). We found incidence of complication in laparoscopic appendectomy group was obviously lower than open appendectomy group, and the difference was statistically significant (χ^2^=9.004, P<0.05). See Table IV.

**Table IV:** Comparison of incidence of complications between two groups [n(%)]

Group	Wound infection	Intestinal obstruction	Intra peritoneal abscess	Incidence of complication (%)
Open appendectomy group (n=130)	8 (6.15)	2 (1.54)	6 (4.62)	12.31
Laparoscopic appendectomy group (n=130)	2 (1.54)	0	4 (3.08)	4.62
X^2^ value				9.004
P value				<0.05

## DISCUSSION

With the constant development and improvement of medical and health facilities and medical technologies, laparoscopic technique tends to show up prominently in clinical diagnosis and treatment and gradually favored by more and more patients.^8^,^9^ A study has proved that, laparoscopic appendectomy characterized by small trauma and rapid recovery can be used in diagnosis and treatment of other diseases and is a preferred surgical method, especially for those who pursue for cosmetic result of incision and obese patients.^10^ But it is also pointed out that, open appendectomy and laparoscopic appendectomy have no differences in aspects of duration of operation and postoperative complications.^11^ But opinions on whether laparoscopic appendectomy is suitable for different types of appendicitis are different. In foreign countries, laparoscopic appendectomy suffered from being questioned to being accepted. In China, laparoscopic appendectomy has not been able to completely replace open appendectomy uptil now, though it has been the preferred surgical method. That is because two kinds of appendectomy have no significant differences in duration of operation and length of stay, and preparation of laparoscopic equipment seems to be too complicated for appendectomy, such as a simple operation; moreover, whether laparoscopic appendectomy is applicable to all kinds of appendicitis is controversial.

Time of operation of open appendectomy and laparoscopic appendectomy is now disputed by some doctors. A previous study^12^ suggests that, laparoscopic appendectomy lasted for a longer time than open appendectomy. For clinical surgery, time of operation is determined by multiple factors, especially the skill level and clinical experience of surgeons.^13^ In this study, operation time of laparoscopic appendectomy (63.48±27.46 minutes) was much shorter than open appendectomy (108.06±51.47 minutes), and there was a remarkable difference (P<0.05). But a previous study points out laparoscopic appendectomy lasted for a longer time compared to open appendectomy, but most recent studies hold the same opinion as in our study.^14^ That is because, most doctors doing surgery were senior doctors with rich experience and high skill level; field of view was wide in laparoscopic surgery, which facilitated searching for appendix. Laparoscopic devices has gained great importance, opening and closing of abdomen could be avoided. With the extensive application of laparoscope in clinical and improvement of skills level of doctors, operation time of laparoscopic appendectomy will no longer be the focal point for discussion.

Blood loss during operation can also have an impact on recovery of patients. In this study, blood loss during operation in laparoscopic appendectomy group (7.94±7.53 mL) was less than open appendectomy group (27.7±38.2 mL) (P<0.05). That is because meso appendix was processed well using ultrasound knife, iron clamp and Hemolock. This is the advantage of laparoscopic appendectomy.

It can be found from experimental results that, clinical indexes (time to leave bed, anus exhaust time, time to take food, catheter drainage time, time of urinary catheterization and application time of antibiotics) of laparoscopic appendectomy group were all superior to open appendectomy (P<0.05), suggesting laparoscopic appendectomy was more beneficial to recovery of patients. Though the application of pain killer differed from two groups, the difference was not significant, suggesting pain caused by both the surgical procedures was the same.

Postoperative complications of patients undergoing appendectomy can be distinguished by severity of appendicitis.^15^,^16^ Postoperative complications of simple appendicitis mainly include incision infection and intestinal injury, while complicated appendicitis includes incision infection, intra abdominal abscess, intestinal obstruction, respiratory tract infection and intestinal adhesion. A previous study suggests that, incision infection and intra abdominal abscess are the major complications occurring after open appendectomy and laparoscopic appendectomy.^17^ It is also found that, incidence of intra-abdominal abscess of patients undergoing laparoscopic appendectomy is higher than that of open appendectomy.^18^,^19^ In this study, incidence of complications of open appendectomy group (12.31%) was much higher than that of laparoscopic appendectomy group (4.62%) (P<0.05), and number of cases of intra-abdominal abscess in laparoscopic appendectomy group was less than open appendectomy group. That is because CO^2^ insufflated in laparoscopic surgery led to spread of bacteria in abdominal cavity, especially in the treatment of perforating appendicitis. To reduce bacteria stay in abdominal cavity, abdominal cavity is thoroughly washed. But it is found afterwards that, washing for large area is helpless, instead it increases risks of pollution of the edge of abdominal cavity. Phlegm suction method was adopted in this study, which lowered incidence of intra-abdominal abscess.

## CONCLUSION

Laparoscopic appendectomy can result in small trauma, rapid recovery, significantly lower incidence of abdominal residual abscess and incision infection as well as good prognosis. But surgical approach still needs to be determined according to disease condition, economic condition, experience of surgeon and condition of the hospital. Though laparoscopic appendectomy has not completely replaced open appendectomy, laparoscopic appendectomy is supposed to be the better option for pediatric appendicitis with the improvement of surgical method and medical technology.
